# Gender Differences in Cardiovascular Risk Profile in Rheumatoid Arthritis Patients with Low Disease Activity

**DOI:** 10.1155/2019/3265847

**Published:** 2019-03-28

**Authors:** Bożena Targońska-Stępniak, Małgorzata Biskup, Wojciech Biskup, Maria Majdan

**Affiliations:** ^1^Department of Rheumatology and Connective Tissue Diseases, Medical University of Lublin, Lublin, Poland; ^2^Wojewódzki Zespół Specjalistyczny, Rzeszów, Poland

## Abstract

**Objective:**

Patients with rheumatoid arthritis (RA) have an excess risk of cardiovascular (CV) disease (CVD). The objective of the study was to compare CV risk profile in female and male RA patients with low disease activity.

**Materials and Methods:**

The study group consisted of 70 RA patients with continuous low disease activity and no CVD (54 women, 16 men) and 33 healthy controls of comparable age. The groups were assessed for blood pressure, serum amino-terminal pro-brain natriuretic peptide (NT-proBNP), carotid intima media thickness (cIMT), electrocardiography, ejection fraction (EF), and diastolic dysfunction (DD).

**Results:**

Significantly higher burden of atherosclerosis, as revealed by higher cIMT, was found in males [0.93 (0.2) mm] vs females [0.80 (0.2) mm]. The risk of 10-year CVD was significantly higher in men than in women with RA. High/very high risk of fatal CVD was found in 62.5% of male patients. Males were significantly more often current/ex-smokers and had lower HDL-cholesterol and higher atherogenic index. There were no significant differences in NT-proBNP, QTc duration, and parameters of EF and DD.

**Conclusions:**

In RA patients with continued low disease activity, a higher burden of atherosclerosis was found in males than in females. The data suggest a significant impact of traditional CV risk factors.

## 1. Introduction

Rheumatoid arthritis (RA) is a chronic autoimmune disease associated with reduced lifespan and excess mortality compared with the general population [1]. The main cause of death is cardiovascular (CV) disease (CVD), accounting for 30-50% of premature deaths observed [2, 3]. Current findings suggest significantly improved overall CV mortality in RA patients in recent years [4]. Beyond traditional risk factors, chronic, systemic inflammation is responsible for the development of accelerated atherosclerosis and heart disease [2, 5–7]. It has been suggested that low disease activity is sufficient to achieve a protective effect against CVD [8, 9].

Many observational studies report that women with RA are affected worse by the disease than men [10–12]. Women report more severe symptoms and greater disability, and disease activity measures seem to be worse in women than in men [11]. It was reported that, in patients with RA, women had a marked increase in mortality from CVD, compared with men [3]. The gender differences in CV risk factors were assessed in the general population; however, little information exists in relation to RA patients [13]. In literature there are no papers assessing CV risk factors in male and female RA patients with low disease activity.

The objective of this study was to assess and compare CV risk profiles in female and male RA patients with continued low disease activity and no history of CV disease.

## 2. Patients and Methods

### 2.1. Patients and Controls

The study was performed on outpatients with RA, treated in the Regional Outpatient Clinic in Rzeszów, Poland, as described previously [14, 15]. The patients fulfilled the American College of Rheumatology (ACR)/ European League Against Rheumatism (EULAR) classification criteria for RA [16].

The protocol of the study was approved by the Ethics Committee of the Medical University of Lublin, with approval number KE-0254/92/2013. The study was performed in accordance with the 1964 Declaration of Helsinki and its later amendments. Informed consent was obtained from each patient after an adequate explanation of the research.

The study group consisted of 70 RA patients, 54 women and 16 men, of comparable age, who had continued low disease activity (4.2 [1.2] years, from two to seven years) and no clinically evident CV [e.g., ischemic heart disease (IHD), heart failure (HF), hypertension], or chronic kidney disease (CKD).

The control group consisted of 33 healthy volunteers (18 women, 15 men).

Demographic and clinical information was obtained through structured interview, review of medical records, self-report questionnaires, physical examination, and laboratory tests. Assessment of the CV system was performed using the following methods: electrocardiogram (ECG), echocardiography, and high-resolution B-mode ultrasonography, as described previously [14, 15].

### 2.2. RA-Related Data Collection

Disease activity of RA was assessed using the Disease Activity Score (DAS) based on evaluation of 28 joints (DAS28), calculated with the number of tender and swollen joints, erythrocyte sedimentation rate (ESR) value, and patient's global assessment of disease activity in the visual analogue scale (VAS) [17]. The cut-off point for low disease activity was DAS28 ≤ 3.2.

Erosive form of RA was diagnosed in those patients who presented with erosions on joint surfaces of bones in radiograms of hands and/or feet, assessed according to the Sharp/van der Heijde score by a trained radiologist [18].

### 2.3. Laboratory Measurements

Blood was collected after overnight fasting. All the tests were performed in the Medical Laboratory Diagnostics of the Regional Outpatient Clinic, according to standardized laboratory methods, as described previously [14, 15].

The standard assessment included complete blood cell count, ESR, serum concentration of C-reactive protein (CRP), glucose, total cholesterol (TC), high-density lipoprotein cholesterol (HDL-C), low-density lipoprotein cholesterol (LDL-C), and triglycerides (TG). The atherogenic index (AI) was calculated as the ratio of TC to HDL-C concentrations (normal value in women <4.0 and in men <4.5).

Blood samples were also taken to assess RA serological markers: rheumatoid factor IgM (RF-IgM) and anti-cyclic citrullinated peptide (anti-CCP) antibodies, using enzyme-linked immunosorbent assays (ELISA): RF-IgM using Johnson&Johnson ELISA assay (normal upper limit 12 IU/ml), anti-CCP antibodies using EUROIMMUN ELISA assay (normal upper limit 5 RU/ml). Serum samples were stored at -80°C for further assessment of amino-terminal pro-brain natriuretic peptide (NT-proBNP).

### 2.4. CV Parameters Assessment

Information concerning CV, diabetes, and CKD was taken from medical records. Blood pressure (BP) was assessed in a sitting position. Height and weight were measured barefoot wearing light clothes. Body mass index (BMI) was calculated as the ratio of weight to squared height.

The Systemic Coronary Risk Evaluation (SCORE) model was estimated to assess the 10-year risk of fatal CVD. The result was multiplied by 1.5 (mSCORE) according to EULAR recommendations [19]. The value ≥5% indicates high risk.

Measurement of NT-proBNP serum concentration was performed using electrochemiluminescence immunoassay, Cobas assay (Elecsys pro-BNP II test); the recommended normal range in patients <75 years is <125 pg/ml.

### 2.5. Electrocardiogram (ECG)

ECG was performed in RA patients and controls, as described previously [14]. The heart rate corrected QT (QTc) was calculated using Bazett's formula (normal QTc value in women <450 ms and in men <430 ms) [20].

### 2.6. Echocardiographic Examination

Echocardiographic examination was performed in RA patients and controls, as described previously [14]. Left ventricle function was assessed by ejection fraction (EF). Early (E) and late (A) diastolic mitral inflow velocities were measured and the E/A ratio was calculated. E/A ratio < 1.0 is equivalent to diastolic dysfunction.

### 2.7. Carotid Intima-Media Thickness (cIMT) Measurement

An assessment of cIMT was performed in RA patients and controls, as described previously [14]. The mean cIMT value <0.6 mm is considered as normal and ≥0.9 mm as abnormal. The cIMT value ≥0.6 mm and <0.9 mm is a marker of subclinical atherosclerosis, and presence of carotid plaques is a marker of advanced atherosclerosis. Plaques were defined as a distinct protrusion of more than 1.5 mm into the vessel lumen [21].

### 2.8. Statistical Analysis

Results were expressed as mean (standard deviation, SD) or number (%) and range of values (minimum and maximum). Variables were tested for normality by the Kolmogorov-Smirnov test. Differences between Rs patients and controls as well as between the specific groups of RA patients were tested using Kruskal-Wallis H-test and Mann-Whitney* U* test, as well as Student's t-test and Chi^∧^2 test, for non-normally and normally distributed parameters, respectively. For all tests *P* values <0.05 were considered significant.

## 3. Results

### 3.1. Demographic and Disease-Related Variables in Female and Male RA Patients

The general characteristics of RA patients (whole group, females and males) are presented in Table 1. Patients included in the study had no history of IHD, HF, hypertension, diabetes, or CKD.

Patients with RA, female and male, were of comparable age. The disease activity was low (DAS28 ≤3.2) in both groups for about four years (from two to seven years). There were no significant differences between females and males regarding disease duration, seropositivity (RF-IgM or anti-CCP), presence of bone erosions and extra-articular manifestations, or the treatment used. There were no patients treated with biological disease-modifying anti-rheumatic drugs (bDMARDs). Conventional synthetic disease modifying anti-rheumatic drugs (csDMARDs) were used in all patients and included methotrexate (MTX) in the vast majority of patients (dose 10-25 mg/week, in monotherapy or combination), leflunomide, and in a few women hydroxychloroquine, sulfasalazine, or cyclosporine A. The mean (SD) dose of MTX in the group of female patients was 18.2 (3.9) mg/week and in male patients 18.3 (3.6) mg/week (difference not statistically significant).Therapy with low-dose glucocorticoid (GK) (prednisone ≤5 mg/day) was used in over 25% of female patients and only in one male patient (difference not statistically significant) (Table 1).

The patients did not take statins or any cardioprotective treatment.

### 3.2. Characteristics of the Control Group

The control group consisted of 33 healthy subjects: 18 women (54.5%) and 15 men (45.5%). They had no traditional CV risk factors. The mean (SD) age was not significantly different in women and in men (52.9 [9.1] and 54.5 [7.5] years, respectively).

No significant difference of the mean cIMT value was observed between women and men in the control group (respectively, 0.63 [0.09] vs. 0.6 [0.12] mm, NS).

### 3.3. Characteristics of CV Parameters in Female and Male RA Patients

The characteristics of CV parameters are presented in Table 2.

The statistically significant differences between male and female patients included lower HDL-C concentration and higher value of AI in males. Men with RA were significantly more often current/ex-smokers. The mean mSCORE value was significantly higher in males. According to the mSCORE system, high or very high 10-year risk of CV death was found significantly more often in men. High/very high mSCORE was observed in over 60% of males with RA and no CV, in comparison with about 25% of female patients (Table 2).

The mean value of cIMT was significantly higher in men than in women with RA. All male patients had increased cIMT, and no men had normal cIMT value (Table 2).

The mean NT-proBNP concentration was significantly higher in female than in male RA patients, but within the normal range (Table 2).

There were no significant differences between female and male patients regarding systolic blood pressure, diastolic blood pressure, BMI, serum glucose, TC, LDL-C, TG concentrations, QTc duration, E/A ratio, and EF (Table 2).

### 3.4. Comparison of CV Parameters in Groups of RA Patients and Controls

The mean age did not differ significantly between patients and controls, both females (53.8 [13.4] and 52.9 [9.1] years, NS) and males (54.1 [12.6] and 54.5 [7.5] years, NS).

**Figure 1:**
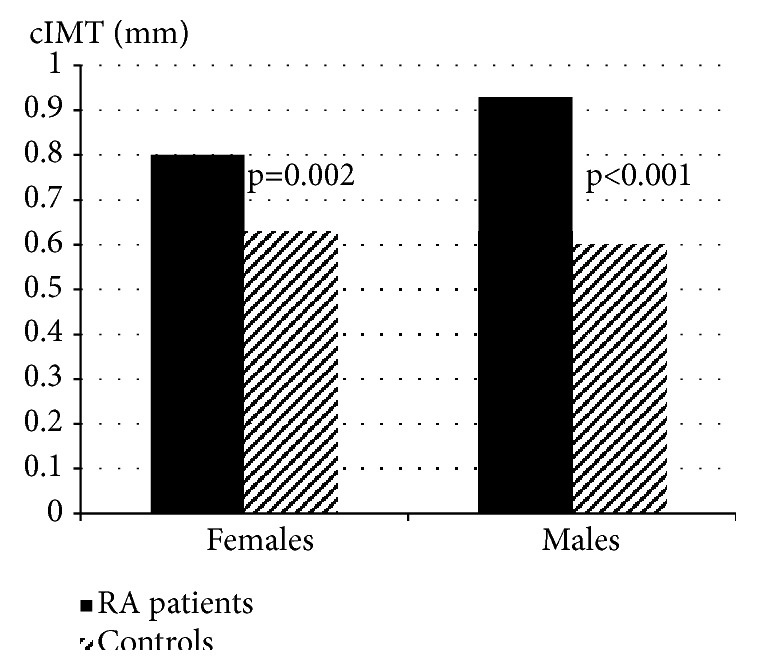
Comparison of cIMT value in RA patients and controls, in females and males.

The comparison of cIMT in patients and controls is presented in Figure 1. The mean cIMT value was significantly higher in male RA patients than in male controls of comparable age, as well as in female patients compared to female controls (Figure 1).

The duration of QTc was significantly higher in female RA patients than in female controls (441.2 [22.9] vs 412.3 [22.6] ms, p<0.0001). There was no such difference between male groups. There were no statistically significant differences between female and male groups of patients and controls in respect to values of E/A ratio and EF.

## 4. Discussion

In this study, in the group of RA patients with continuous low disease activity and no CVD, higher burden of atherosclerosis and significantly higher risk of CV death were found in male patients. To the best of our knowledge, this is the first report concerning gender differences of CV risk parameters in RA patients who had continued low disease activity.

The higher burden of atherosclerosis in male patients was revealed by significantly greater cIMT compared with female RA patients, and also with male healthy controls, both of comparable age. According to the data in the literature, on average cIMT is greater in men than in women and increases with age [22]. However, in this study the mean cIMT value was not significantly different in male and female healthy controls.

The higher 10-year risk of CV death according to mSCORE was found in men with RA, despite comparable age of men and women; however, men were significantly more often current/ex-smokers. It is noteworthy that high/very high risk of CV death according to mSCORE was noted in over 60% of male patients with RA, despite continued low disease activity and no history of CVD.

Significantly higher NT-proBNP concentrations were observed in female than in male RA patients, which is consistent with the data in the literature. It has been reported that NT-proBNP values are substantially higher in women compared with men at every age, and levels increase with increasing age for both sexes [23].

The higher burden of atherosclerosis was found in both male and female patients with RA in comparison with respective male and female controls of comparable age, according to the significantly higher cIMT in RA patients. The significantly longer QTc duration found in female patients than controls might suggest higher risk of sudden CV death. According to the literature, variables of QTc interval are significantly increased in RA patients compared with healthy controls, and QTc prolongation is a strong predictor of death, independently associated with all-cause mortality in RA [24].

Patients with RA, compared with the general population, have an increased risk of CVD, which is probably related to both traditional CV risk factors and RA-specific characteristics, particularly inflammatory disease activity [1, 2, 13]. Inflammation may also influence traditional cardiovascular risk factors, such as lipoproteins [13]. Systemic inflammation associated with disease activity seems to account for a large proportion of the increased CV risk in RA patients [3, 13]. Effective disease-modifying treatment, by suppressing inflammation, may reduce the risk of CVD [3, 5]. The beneficial effect of low disease activity has been reported in patients with RA [8, 9]. Data in the literature indicate that atherosclerosis is not accelerated in RA of low activity or remission [8]. In this study, a higher burden of atherosclerosis was observed in RA patients despite continued low disease activity.

The data on gender differences in RA are inconsistent; some data suggest less favorable course of the disease in men, while many other studies indicate less favorable status in women [10, 11]. In our study, female and male patients had comparable disease activity and severity (incidence of erosions, extra-articular manifestations, antibodies).

In the general population, differences between the sexes occur in risk factors and clinical manifestations of CVD. The INTERHEART study found that smoking had a higher impact on CVD in men than in women, and hypertension and diabetes had a higher impact in women than in men [25]. However, in another study, higher impact of smoking was found in women than in men [26]. In a recent study evaluating a large international cohort of RA patients with different levels of disease activity, no significant sex differences were observed concerning the effect of risk factors (traditional and RA-specific) on future CVD. A total of 70% of CV events were attributable to all CV risk factors and 30% of CV events were attributable to RA characteristics [13]. In this study, unfavorable traditional CV risk factors (smoking, lower HDL-C, higher AI) and higher mSCORE were found significantly more often in male than in female patients, despite the comparable age. It seems that, in RA patients with continuous low disease activity, traditional risk factors have a significant impact on CVD risk progression.

The strengths of this study include the homogeneous study population, which consisted of patients with continued low disease activity and simultaneously no history of CVD or other diseases that increase CV risk; all the patients were treated with csDMARDs, and there were no patients with biological treatment; all assessments of CV parameters were performed by an experienced cardiologist.

The limitations of the study include the following: the number of patients was low (a higher number of patients would enable more accurate statistical analysis); use of ultrasound examination of joints or nontraditional serological inflammatory markers could allow more precise assessment of the inflammatory state and disease activity in patients with low activity according to DAS28.

## 5. Conclusions

The significantly higher burden of atherosclerosis was found in male RA patients compared with female RA patients, despite comparable age and continued low disease activity (DAS28 ≤3.2). The data suggest the significant impact of traditional CV risk factors resulting in high or very high risk of fatal CVD in most male RA patients who have low disease activity.

## Figures and Tables

**Table 1:** Characteristics of 70 patients with RA and without CVD.

Characteristics	Overall (n=70)	Female (n=54)	Male (n=16)	p (F vs. M)
Age, years	53.9 (13.1)	53.6 (12.4)	54.3 (10.3)	0.8
RA related variables:				
Disease duration, years	6.9 (3.3)	7.0 (3.4)	6.3 (3.1)	0.4
Low RA activity duration, years	4.2 (1.2)	4.2 (1.2)	4.0 (1.0)	0.6
Positive RF-IgM	54 (77.1)	41 (75.9)	13 (81.3)	0.7
Positive anti-CCP	53 (75.7)	40 (74.1)	13 (81.3)	0.6
Erosions (hands/feet)	37 (52.9)	28 (51.9)	9 (56.3)	0.8
Extra-articular manifestations	14 (22.9)	12 (22.2)	2 (12.5)	0.4
DAS28	2.87 (0.2)	2.88 (0.2)	2.83 (0.23)	0.4
CRP, mg/l	8.7 (12.9)	7.9 (6.8)	11.4 (24.3)	0.3
ESR, mm/h	14.8 (10.2)	15.8 (10.1)	11.3 (9.7)	0.1
Treatment:				
Current glucocorticoid use	16 (22.8)	15 (27.8)	1 (6.3)	0.07
Current conventional DMARD	70 (100)	54 (100)	16 (100)	
MTX monotherapy	47 (67.1)	33 (61.1)	14 (87.4)	0.3
LEF monotherapy	11 (15.7)	10 (18.5)	1 (6.3)	
HCQ monotherapy	5 (7.2)	4 (7.4)	0	
SS monotherapy	1 (1.4)	1 (1.9)	0	
CsA monotherapy	1 (1.4)	1 (1.9)	0	
DMARDs combination	5 (7.2)	5 (9.2)	1 (6.3)	

Data are presented as mean (SD) (range) or number (%).

Abbreviations: anti-CCP, anti-cyclic citrullinated peptide antibodies; CRP, C-reactive protein; CVD, cardiovascular disease; CsA, cyclosporine A; DAS28, disease activity score in 28 joints; DMARD, disease modifying antirheumatic drug; ESR, erythrocyte sedimentation rate; HCQ, hydroxychloroquine; LEF, leflunomide; MTX, methotrexate; RF-IgM, IgM rheumatoid factor; SS, sulfasalazine.

**Table 2:** Cardiovascular parameters in 70 patients with RA and without CVD.

Characteristics	Overall (n=70)	Female (n=54)	Male (n=16)	p (F vs. M)
Traditional CVD risk factors				
SBP, mmHg	128.3 (9.4)	128.1 (9.9)	128.8 (7.4)	0.8
DBP, mmHg	81.0 (7.4)	80.5 (7.8)	82.8 (5.5)	0.3
Serum glucose, mg/dl	91.9 (5.2)	92.0 (5.3)	91.5 (4.9)	0.7
BMI, kg/m^2^	25.5 (3.2)	25.2 (4.1)	25.5 (3.2)	0.8
Total cholesterol, mg/dl	196.7 (38.9)	195.6 (35.7)	200.6 (49.6)	0.7
HDL cholesterol, mg/dl	57.0 (14.5)	59.0 (14.6)	50.2 (12.0)	0.03*∗*
LDL cholesterol, mg/dl	116.8 (35.7)	114.6 (32.9)	124.0 (44.1)	0.4
Triglycerides, mg/dl	113.6 (55.4)	109.3 (39.9)	128.1 (90.4)	0.2
Atherogenic index	3.6 (1.2)	3.4 (1.0)	4.2 (1.4)	0.03*∗*
Non-smoker	63 (90.0)	52 (96.3)	11 (68.8)	0.001*∗*
Current/ Ex-smoker	7 (10.0)	2 (3.7)	5 (31.2)	0.001*∗*
mSCORE, %	3.43 (3.19)	2.76 (2.73)	5.69 (3.68)	<0.001*∗*
High/Very high risk of CV death	23 (32.9)	13 (24.1)	10 (62.5)	0.004*∗*
Ultrasonography of carotid arteries				
cIMT, mm	0.83 (0.21)	0.80 (0.2)	0.93 (0.2)	0.04*∗*
Normal cIMT (<0.6 mm)	10 (14.3)	10 (18.5)	0	0.6
cIMT ≥ 0.6 and <0.9 mm	34 (48.6)	26 (48.2)	8 (50.0)	0.9
Arterial wall hypertrophy >0.9 mm	26 (37.1)	18 (33.3)	8 (50.0)	0.2
Echocardiography				
E/A ratio	1.08 (0.28)	1.1 (0.28)	1.03 (0.29)	0.4
EF, %	59.8 (1.6)	60 (0.97)	59.1 (2.72)	0.5
Electrocardiogram: QTc, ms	439.6 (23.7)	441.2 (22.9)	433.9 (26.0)	0.3
NT-proBNP, pg/ml	97.6 (63.4)	106.8 (61.5)	66.6 (61.2)	0.02*∗*

Values are presented as mean (SD) (range) or number (%).

Abbreviations: BMI, body mass index; cIMT, carotid intima media thickness; CVD, cardiovascular disease; DBP, diastolic blood pressure; EF, ejection fraction; HDL cholesterol, high-density lipoprotein cholesterol; LDL cholesterol, low-density lipoprotein cholesterol; mSCORE, multiplied systemic coronary risk evaluation; NT-proBNP, amino-terminal pro-brain natriuretic peptide; SBP, systolic blood pressure.

## Data Availability

The demographic and history data used to support the findings of this study have been deposited in the Malgorzata Biskup repository. The clinical and laboratory data used to support the findings of this study are included within the article.
